# Topological image modification for object detection and topological image processing of skin lesions

**DOI:** 10.1038/s41598-020-77933-y

**Published:** 2020-12-03

**Authors:** Robin Vandaele, Guillaume Adrien Nervo, Olivier Gevaert

**Affiliations:** 1 Stanford Center for Biomedical Informatics Research (BMIR), Department of Medicine, Stanford University School of Medicine, Stanford, CA 94305 USA; 2 Department of Biomedical Data Science, Stanford University School of Medicine, Stanford, CA 94305 USA; 3 IDLab, Department of Electronics and Information Systems, Ghent University, Technologiepark-Zwijnaarde 19, 9052 Gent, Belgium; 4 Data mining and Modelling for Biomedicine (DaMBi), VIB Inflammation Research Center, Technologiepark-Zwijnaarde 927, 9052 Gent, Belgium; 5 Institute for Computational and Mathematical Engineering, Stanford, CA 94305 USA

**Keywords:** Medical research, Mathematics and computing

## Abstract

We propose a new method based on *Topological Data Analysis* (TDA) consisting of Topological Image Modification (TIM) and Topological Image P*rocessing* (TIP) for object detection. Through this newly introduced method, we artificially destruct irrelevant objects, and construct new objects with known topological properties in irrelevant regions of an image. This ensures that we are able to identify the important objects in relevant regions of the image. We do this by means of *persistent homology*, which allows us to simultaneously select appropriate thresholds, as well as the objects corresponding to these thresholds, and separate them from the noisy background of an image. This leads to a new image, processed in a completely unsupervised manner, from which one may more efficiently extract important objects. We demonstrate the usefulness of this proposed method for topological image processing through a case-study of unsupervised segmentation of the ISIC 2018 skin lesion images. Code for this project is available on https://bitbucket.org/ghentdatascience/topimgprocess.

## Introduction

For the past decade, *persistent homology*^1^—the most prominently used and studied tool within the field of *Topological Data Analysis* (TDA)^2^—has lead to many new applications for image segmentation and classification^3–8^. The great potential of TDA for images is due to their grid-like structure. In this way, one may study topological properties of the depicted object(s) through persistent homology, which *tracks topological changes of a changing space*, without the need of an intermediate combinatorial structure such as the *Vietoris-Rips complex* for point cloud data^9^.

**Figure 1:**
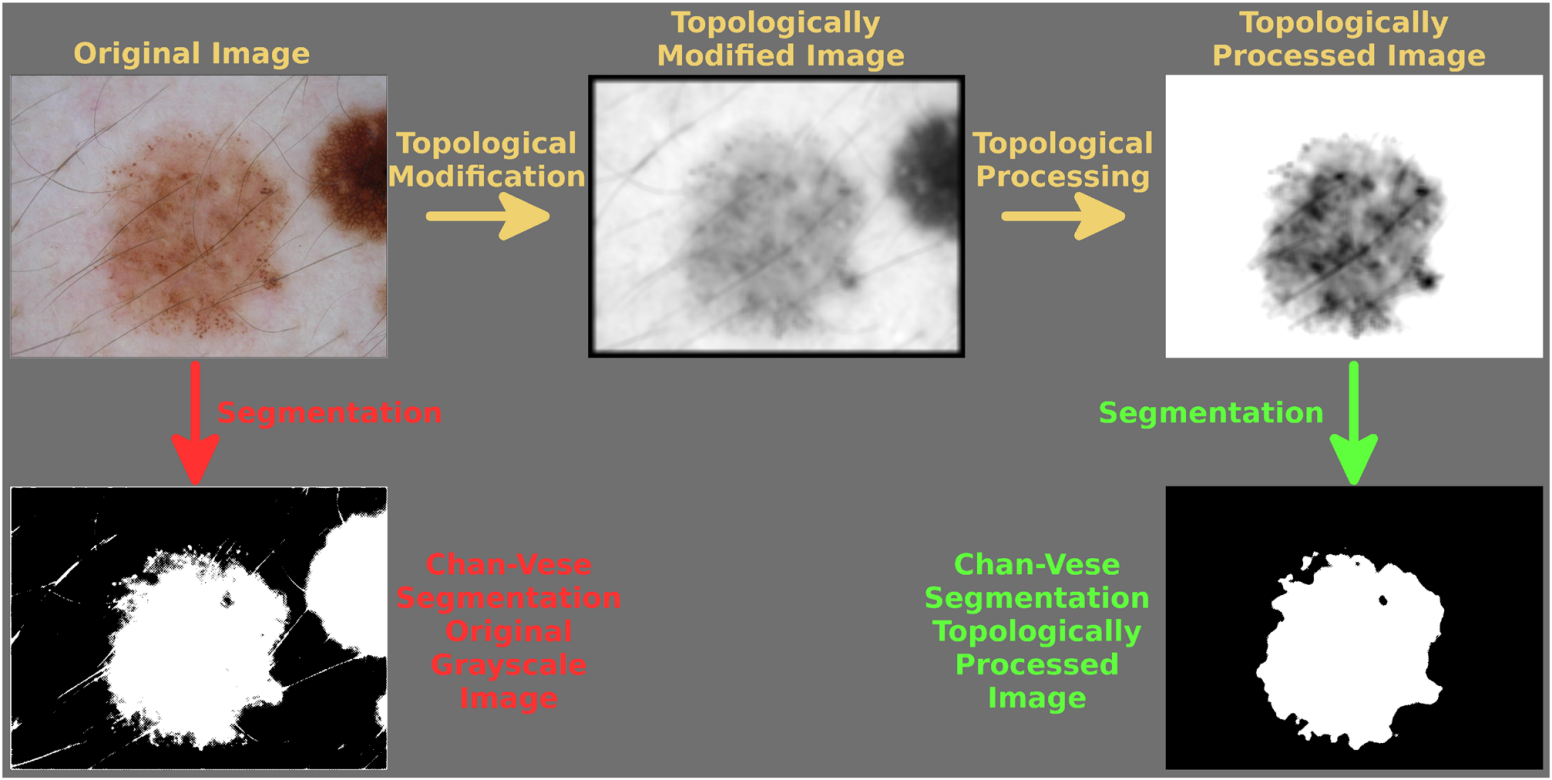
An overview of how topological image modification and processing improves the performance of the completely unsupervised Chan–Vese segmentation algorithm^10^. Lesion image from ISIC 2018^11,12^.

Our current work builds upon the idea that persistent homology can be used to detect objects in images^5^. However, many real-world images contain outliers, as well as irrelevant objects, which complicate the use of persistent homology for this purpose. We enclose this gap by introducing Topological Image Modification (TIM). TIM targets improving Topological Image Processing (TIP)—processing an image based on the aggregation of its topological information—by *filtering out significant but irrelevant topological information*. We consider the use of TIP for enhancing the ability to identify and segment important objects on images, increasing the performance of existing models and algorithms for this purpose.

Unlike existing TDA methods for object detection or segmentation, through TIM and TIP we are able to discard significant but irrelevant objects^5,7^, which is the main purpose for which we designed this method. Furthermore, although we will consider skin lesion images for illustrating the effectiveness of our method, we do not require any specific textural assumptions restricted to this domain^7^. We also mark the relevant objects in images in a robust manner, rather than producing a parameter sensitive oversegmentation of the entire image^8,13^. Finally, we may pass our resulting processed image to any segmentation algorithm, and do not target active contour based segmentation methods in particular^8^.

Our case-study will consider skin lesion images^14,15^, ideal for illustrating the intuition behind our method. Nevertheless, TIM (and as result, TIP) generalizes to many types of real-world images through its generic assumptions, without requiring any supervision^16,17^. Furthermore, although any existing method for segmentation or object detection (or that might be modified for this purpose)^10,18–22^ could lead to a possibly generic method for TIP as a replacement of Algorithm 1 which we introduce in this work, we will make clear that these are unfit for this purpose in our experiments. To the best of our knowledge, neither TIM, the concept of TIP, nor the flow ‘TIM $\rightarrow$ TIP $\rightarrow$ Segmentation’ has been introduced or studied before.

Figure 1 illustrates an example of our proposed approach. The original image depicts a centered skin lesion (the relevant object of this image). The image also depicts other irrelevant objects, such as strands of hair and a part of another lesion connecting to the border of the image. Both the irrelevant and relevant objects, as well as the border of the image, are all included in the result of the *Chan–Vese* segmentation algorithm (an unsupervised segmentation algorithm for single channel images^10^) on the corresponding grayscale image. However, the same segmentation algorithm on the topological processed image provides a much better segmentation, that only includes the relevant object. This processed image was obtained from the topological information of our topologically modified image. In the following sections, we describe our approach in more detail. We emphasize that we introduce TDA as a new method for object detection using skin lesions segmentation as an application.

The main contributions of this work are as follows. First, we show the existing difficulties of TDA for object detection in real-world images, and introduce *topological image modification* (TIM) to overcome these. Next, we show how image smoothing can be regarded as a *destructive* way of TIM, and introduce a new and more *constructive* way, i.e, *border modification*. Following this, we show how TIM leads to a powerful new method for *topological image processing* (TIP), for which we introduce a new algorithm that marks objects in an image consistent with the (number of) inferred components from its *persistence diagram* (Algorithm 1). We demonstrate how TIM and TIP effectively improves six different generic unsupervised models and algorithms through a case-study of the ISIC 2018 skin lesion images in our experiments. Finally, we summarize how our method leads to and opens up new possibilities for TIP.

## Material and methods

### Skin lesion images

We consider 2594 skin lesion images from the ISIC 2018 data set^11,12^. The relevant object on each image was a skin lesion, for which a ground truth segmentation was available. This data set can be obtained through https://challenge2018.isic-archive.com/.

### Persistent homology of images

Persistent homology has its roots in the field of *algebraic topology*^23,24^. Its computation requires two things: a *simplicial complex* *K*, and a *filtration* ${\mathcal {F}}$ defined on *K*. A simplicial complex can be seen as a generalization of a graph, that apart from 0-simplices (nodes) and 1-simplices (edges), may also include 2-simplices (triangles), 3-simplices (tetrahedra), up until *k*-simplices, where $k\in {\mathbb {N}}$ is the *dimension* of the complex. A simplicial complex *K* is furthermore closed under inclusion, i.e., if $\sigma '\subseteq \sigma \in K$ then $\sigma '\in K$. A filtration ${\mathcal {F}}$ on *K* is then a nested sequence $K_0\subseteq K_1\subseteq \ldots \subseteq K_N=K$ of subcomplexes of *K*.

**Figure 2:**
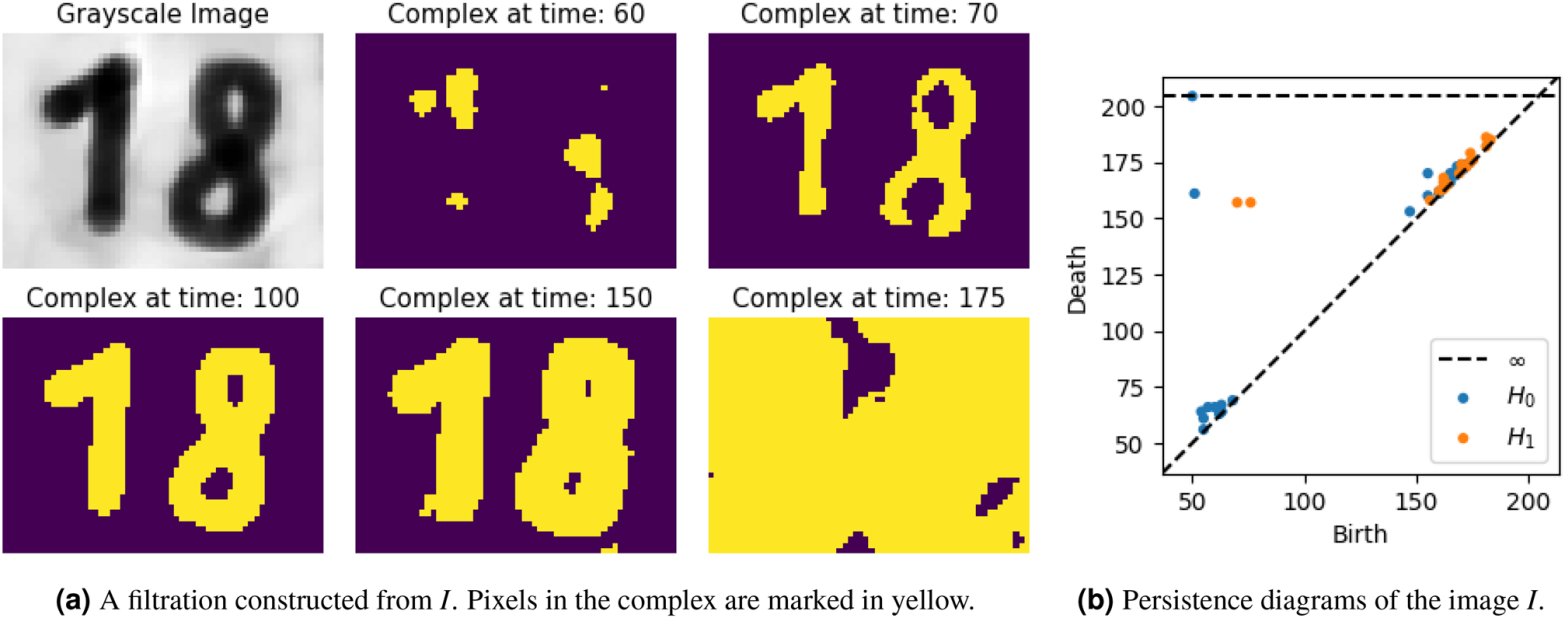
Persistent homology of a grayscale image *I*. Two ‘outlying’ lifetimes for 0-dimensional holes ($H_0$) represent the two components of *I* (the ‘1’ and ‘8’). Similarly, the two ‘outlying’ lifetimes for 1-dimensional holes ($H_1$) represent the two holes in *I* (the holes in the ‘8’).

Given an $M\times N$ image *I*, a simplicial complex *K* naturally follows from the pixel configuration of *I*. More specifically, we define *K* by connecting each pixel to its eight (horizontally, vertically, and diagonally) neighboring pixels. This results in a 1-dimensional simplicial complex (a graph), only including nodes (pixels) and edges. A 2-dimensional simplicial complex can then be obtained through filling in all triangles of this complex. We then define the scalar function

$$\begin{aligned} f:K\rightarrow {\mathbb {R}}:\sigma \mapsto \max _{p\in \sigma }{\text {gray}}_I(p), \end{aligned}$$

where ${{\text {gray}}}_I(p)$ denotes the grayscale value

1 $$\begin{aligned} {{\text {gray}}}_I(p) = \frac{1}{1000}\left( 299{\text {R}}_I(p) + 587{\text {G}}_I(p) + 114{\text {B}}_I(p)\right) \end{aligned}$$

given to pixel *p* in the RGB image *I*. Note that this is just the standard linear converter implemented in the PIL library in Python. The filtration ${\mathcal {F}}$ for *I* is then defined as the *sublevel filtration*

$$\begin{aligned} {\mathcal {F}}:=(\{\sigma \in K:f(K)\le t\})_{t\in {\mathbb {R}}}. \end{aligned}$$

Note that ${\mathcal {F}}$ only changes at finitely many steps, so that we may assume ${\mathcal {F}}$ equals a filtration $K_0\subseteq K_1\subseteq \ldots \subseteq K_T=K$ for some unspecified $T\in {\mathbb {N}}$. Defining the image $I_j$ as having a pixel value of 1 if the corresponding pixel is in $K_j$, and 0 otherwise, a connected component in $K_j$, $j\in \{0,1,\ldots ,T\}$, is then a maximal connected cluster of pixels with every value equal to 1. This filtration is illustrated in Fig. 2a.

The number of *k*-dimensional holes in a complex is expressed through the *Betti number* $\beta _k$. In this sense, a 0-dimensional hole is a ‘gap’, and $\beta _0$ corresponds to the number of connected components, and $\beta _1$ corresponds to the number of loops, $\beta _2$ to the number of voids, and so on. However, in the case of (2D) images, we may only discover up to 1-dimensional holes.

Persistent homology quantifies topological changes through the *birth* and *death* of these holes across the filtration, and is invariant to rotation, translation, or warping of the image^25^. The idea behind persistent homology and persistence is that holes persisting for a long range of consecutive values *t* represent significant features of the topological objects depicted by the image. This is illustrated by the *persistence diagrams* ${\mathcal {D}}_k$ (one for each considered dimension $k\in \{0,1\}$ of holes) in Fig. 2b. This is a multiset containing a point (*b*, *d*) for each component or hole that was born at $t=b$ and died at $t=d$. By definition, $d=\infty$ if a component or hole never dies. These points are usually displayed at the top of the diagram. Furthermore, by convention, a persistence diagram contains every point on the first diagonal. This ensures that for two diagrams ${\mathcal {D}}$ and ${\mathcal {D}}$, the *bottleneck distance*

$$\begin{aligned} d_{\text{b}}\left( {\mathcal {D}}, {\mathcal {D}}'\right) :=\inf _{\varphi }\sup _x\Vert x-\varphi (x)\Vert _{\infty }\in {\mathbb {R}}_{\ge 0}\cup \{\infty \}, \end{aligned}$$

where $\varphi$ ranges over all bijections from ${\mathcal {D}}$ to ${\mathcal {D}}'$, is well-defined (they both have cardinality $|{\mathbb {R}}|$, and by convention $\infty -\infty =0$). Unless certain pixels are excluded from the image, e.g., they are transparent, there will always be one connected component having an infinite lifetime for the image. Bottleneck distances between images without excluded pixels are always finite.

The *elder rule* states that when constructing the persistence diagram, if two components or holes are merged, by convention, the youngest of them, i.e., the one with the greatest birth-time, dies. Hence, the component with infinite persistence is born at the lowest value that the function defining the filtration (in our case the grayscale value) reaches on the image.

Figure 2b shows the persistence diagram for the filtration in Fig. 2a. We see that two connected components ($H_0$) have a significantly longer lifespan than all other points. This corresponds to the ‘1’ and ‘8’ component of the original image. The two significantly longer lifespans for the 1-dimensional holes correspond to the two holes in the ‘8’ component. For object detection in images through persistent homology, we will only consider the connected components.

It is a well-known and important fact that topological persistence is *stable* under noise^26^. More formally, if *I* and *J* are two images of the same dimension, then

$$\begin{aligned} d_{\text{b}}\left( {\text {Dgm}}(I), {\text {Dgm}}(J)\right) \le \Vert f-g\Vert _{\infty }, \end{aligned}$$

where the filtrations resulting in the diagrams of *I* and *J* are defined through scalar functions *f* and *g*, such as the grayscale value above. Intuitively, this means that if there is only a small pixel-wise difference between the values of *f* and *g*, then their resulting diagrams will be close according to the bottleneck distance. This result is furthermore important to our current work, as our chosen implementation based on the Ripser library in Python for computing persistence does not allow to track the pixels through which particular components are born or die^27^. However, if each pixel in our image has a unique value, we can just match the birth time of a component to the corresponding pixel in the image. Hence, in practice, we apply a small amount of random noise to our image for this purpose. Due to the stability of persistence diagrams, this will not affect the performance of our method. Other implementations that do allow to track birth and death pixels can e.g. be found in the Dionysus 2 library in Python. Note that Algorithm 1 which we present below requires one of these methods to match diagram points to birth and death pixels.

### Topological image modification

We introduce two types of topological image modifiers.

#### Image smoothing

The first type of method for TIM we consider is *image smoothing*. Note that this method has previously been used in conjuction with persistent homology of images^5^. However, its true potential within the context of TIM remained unnoticed. For each pixel *p* of an image *I* and $k\in 2{\mathbb {N}}+1$, we may consider a $k\times k$ square pixel neighborhood ${\mathcal {N}}_k(p)$ centered at *p*, and restricted to *I*, i.e., undefined beyond its borders. We then define a new image $I'$ from *I* by averaging over the neighborhood ${\mathcal {N}}_k(p)$ for each pixel *p*. We observe that a value of $k\sim \Delta (I) / 25$, where $\Delta (I)$ denotes the diagonal length of image *I* in pixels, provides effective results, and that our method which we discuss below is robust against this choice of parameter.

#### Border modification

In the case of real-world images, some may have borders, some may have irrelevant objects, and some may only display the actual objects of interest. Hence, in a generic setting, it becomes difficult to guarantee that the most persisting components correspond to the most important objects of an image, without prior information on their location in the image. Instead, we apply a simple, intuitive, yet powerful ‘trick’. More specifically, through TIM, we guarantee that the most persisting component does *not* correspond to the important object(s) in the image.

*Border modification* builds upon this idea through the generic property that in many real-world images, the object(s) of interest do not connect to the border of the image, but the background does. Note that this is a strictly weaker assumption than assuming that the object(s) of interest are near the center of the image. More formally, border modification constructs a new image $I_b$ from an image *I*, by ensuring that every pixel within a distance *l* of the border of $I_b$ reaches the lowest value, while other values remain unchanged. Due to the elder rule, this ensures that every object connecting to this border will be born through the border. Hence, all of these irrelevant components correspond to the single point with infinite persistence in the persistence diagram of $I_b$. In this way, we are able to restrict the analysis of our persistence diagram for identifying objects to the points corresponding to components with finite persistence. We observe that a value of $l\sim \Delta (I) / 100$ provides effective results, and that our method which we discuss below is robust against this choice of parameter.

### Topological image processing

*Topological image processing* (TIP) means that we process images based on this topological information. Our first step is to decide how many components are displayed by the image, through the distribution of the lifetimes. Note that we may restrict the diagram to only include finite lifetimes by applying topological image (border) modification. Without supervision, any standard outlier detection tool may be used to select such thresholds. However, we will use a method previously described in^28^. This method is based on the result that relevant peaks (of the function defining the filtration) can be extracted from the persistence diagram if it contains a band of a certain width that does not contain any points^29^. More specifically, we look for the the largest empty region parallel to the diagonal we can draw into the persistence diagram. To achieve this, we simply iterate over all lifetimes in decreasing order, and track the difference between consecutive lifetimes. A threshold $\tau$ is then obtained by taking any $\tau$ between the two lifetimes where the largest of these differences is achieved. This procedure is especially useful in conjunction with TIM. If any consecutive difference in the ordered lifetimes would be infinite, it would always be selected.

Once a threshold $\tau$ has been selected, we process our images as to increase the contrast between the objects with a lifetime above $\tau$, and the rest (the background) of the image. For this, observe that if any component with birth-time *b* and lifetime *L* dies through another component, due to the elder rule, the latter component has birth-time $b'\le b$ and lifetime $L'\ge L$. This means that if any component is identified to be significant, the component causing its death is as well. This observation implies thatAlgorithm 1 provides a binary image, marking objects of the original image consistent with (the number of) inferred components through its persistence diagram.

Finally, we apply multivariate interpolation to fill in the background pixels. More formally, for every pixel *p*, we determine the closest pixel $p_1,\ldots ,p_k$, in each of the *k* identified components. *p* is then assigned to an interpolation of the values of pixels $p_1,\ldots , p_k$, by means of inverse distance weighting^30^. By applying this on our topological modified (smoothed) image, we obtain a smooth transition between our object(s) and the background, as well as between different parts of the background.

Runtime Analysis of Topological Image Processing Assuming a (topologically modified) image *I* consisting of $n = M \times N$ pixels, 0-dimensional persistent homology can be computed in ${\mathcal {O}}(n\alpha (n))$ time using a union-find structure^31^. Here $\alpha (\cdot )$ is the inverse of the Ackermann function, which for all practical purposes may be considered a constant no greater than 4^32^. If ${\mathcal {D}}$ is the resulting persistence diagram of *I*, any outlier detection method can be used to infer the number of components *c*. Our chosen method described by^28^ runs in ${\mathcal {O}}(|{\mathcal {D}}|\log |{\mathcal {D}}|)$ time, where $|{\mathcal {D}}|$ is the number of nonzero lifetimes displayed by the diagram, and generally much smaller than *n* for images. Consequently, the *c* objects in the image may be marked in ${\mathcal {O}}(cn)$ time (Algorithm 1). Note that *c* is also significantly smaller than $|{\mathcal {D}}|$ (equalling the number of ‘outlying points’ in ${\mathcal {D}}$), and hence, than *n*. Remaining pixels can then be filled in in ${\mathcal {O}}(cn)$ time through inverse distance weighting^33,34^, resulting in the final topologically processed image. In summary, the total computational complexity of topologically processing an image of *n* pixels is ${\mathcal {O}}(n(\alpha (n) + \log n + c))$, where *c* is the (usually very small) number of inferred components.

### Unsupervised models for ISIC skin lesion

We will investigate the applicability of six different generic unsupervised models and algorithms to ISIC 2018 skin lesion images before TIP, after smoothing only, and after TIP.

#### Chan–Vese segmentation

First, we will consider the Chan–Vese segmentation algorithm^10^. This is a very generic segmentation algorithm designed to segment objects without clearly defined boundaries, not particularly targeted towards skin lesion, or even biased towards darker objects. The algorithm is based on level sets that are evolved iteratively to minimize an energy function. We used the standard settings of the algorithm implemented in the scikit-image library in Python.

#### ISODATA threshold segmentation

Next, we will consider an unsupervised threshold segmentation where the segmentation is composed by the pixels with a value below, i.e., darker than a certain threshold. The threshold was selected based on the ISODATA method^18^, using the standard settings of the algorithm implemented in the scikit-image library in Python.
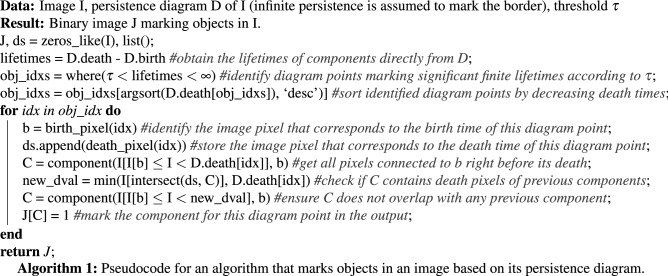


#### Isocontour segmentation

We furthermore consider a segmentation algorithm based on identifying **isovalued contours** in the image, i.e., contours in the image where the pixel value remains constant. For this, we use a special case of the marching cubes algorithm implemented in the scikit-image library in Python^19^. The final segmentation is then obtained by filling in the obtained contours—regarded as polygons in the Euclidean plane—using the OpenCV library. This method differs from a threshold segmentation, in that the isovalued contours are always closed (unless they intersect the border of the image), and that lighter patches enclosed by the contour(s) will also be filled in. The main hyperparameter is the constant which the isocontours should value to. We will simply consider the mean value of the image for this purpose.

#### Clustering based segmentation

Superpixel segmentation algorithms use clustering algorithms in the color space to produce *oversegmentations* (more segments than necessary), and are generally less effective when straightforwardly applied for the task of binary segmentation. Different segments (clusters) in the result are referred to as *superpixels*. We will use a *k*-means clustering based superpixel segmentation algorithm^20^ to segment skin lesion images in 20 different superpixels.

#### Edge detection

Edge detection methods searches for linear segments that correspond to edges and borders in an image. They differ from segmentation methods in that they do not target the output of well-defined (2D) areas. We will investigate the applicability of *Roberts’ cross operator* for edge detection^21^ to skin lesion images.

#### Active contour segmentation

The active contour model is a method to fit open or closed splines, referred to as *snakes*, to lines or edges in an image^22^. It is based on the minimization of an energy function, similarly to the Chan–Vese segmentation algorithm. This method leads to a straightforward binary segmentation by taking the interior of the resulting snake. It requires an initial estimate surrounding the object of interest, making it difficult to apply to the original (skin lesion) images in a consistent and effective way, without additional supervision. However, through TIM and TIP, we are able to provide both an effective initialization and segmentation. More specifically, Algorithm 1 marks a surrounding area for each object of interest, containing no other significant objects. Hence, we use the convex hull of each area marked by Algorithm 1 to initialize the active contour segmentation algorithm.

### Evaluating TIP for ISIC 2018 skin lesion segmentation

The Chan–Vese segmentation, ISODA threshold segmentation, and Isocontour segmentation straightforwardly lead to binary segmentations for both the original skin lesion images, as well as the topologically processed images. We will convert each to grayscale to construct the scalar filtration function (1), after which we apply random normal noise ($\sigma ^2=0.01$), and topologically modify each image through smoothing and border modification. Note that the purpose of the addition of noise is not to affect our performance, but to identify birth-pixels, as discussed above. For each image *I* with diagonal length $\Delta (I)$, we set the smoothing parameter $k\sim \Delta (I) / 25$ and the border width $l\sim \Delta (I) / 100$, while satisfying the integer requirements. Topological image processing is then performed on the topological modified images.

By marking pixels included in the segmentation as positives, we quantitatively evaluate segmentations for all 2594 skin lesion images before TIP, after smoothing, and after TIP, through the metrics listed below.

- The *Accuracy*
  $$\begin{aligned} \frac{TP + TN}{TP + TN + FP + FN}\in [0,1], \end{aligned}$$
  a common validation metric for binary classification.
- The *Sørensen-Dice Coefficient*
  $$\begin{aligned} \frac{2TP}{2TP + FP + FN}\in [0,1], \end{aligned}$$
  a statistic assessing the similarity of two samples.
- *Matthews correlation coefficient*
  $$\begin{aligned} \frac{TP * TN - FP * FN}{(TP+FP)(TP+FN)(TN+FP)(TN+FN)}\in [-1,1], \end{aligned}$$
  measuring the correlation between truth and predicted.
- The *Inclusion Score (also known as the recall)*
  $$\begin{aligned} \frac{TP}{TP + FN}\in [0,1], \end{aligned}$$
  assessing how well the predicted encompasses the truth.

## Results

### Peristent homology of skin lesion images

**Figure 3:**
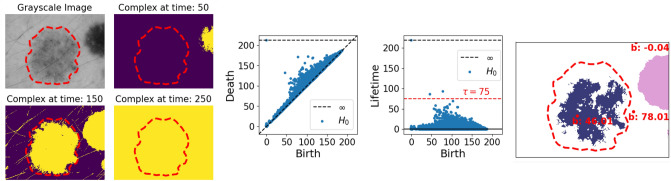
(*Left*) Three complexes $K_t$ at different time steps in the original image. (Middle). The resulting persistence diagram and lifetimes obtained by rotating the diagram. (*Right*) The identified components—those with lifetimes above the thresholds marked by the red striped line—right before their lowest death-time, as well as their birth-pixel and value. Ground truth segmentation borders are marked in red on all images. Lesion image from ISIC 2018^11,12^.

Figure 3 shows a skin lesion image *I* that contains multiple *true* objects: strands of hair, (part of) a non-relevant lesion connecting to the boundary, and a centered lesion: the object of interest. Since these objects are darker than the skin tissue, they should correspond to persisting components in the sublevel filtration of *I*. Hence, a first attempt to identify the important objects of *I*, is through the points in its persistence diagram marking components with a high persistence (Fig. 3). Note that apart from the single component with infinite persistence, which will always be present for images, we also note two other relatively long persisting components, with a lifetime above 75. Including the component with infinite persistence, these correspond to three components that are all alive right before the lowest of their death-times (Fig. 3).

This example illustrates the first problems that arises from applying topological persistence as a method for object detection in real-world images. Though topological persistence is stable in terms of noise, it is not robust to outliers. In the case of images, this means that a single or insignificant cluster of pixels can be identified as a significant component through persistent homology. This is the case for the component born at time $\sim$78 in Fig. 3 (Right). Furthermore, persistent homology also identifies true but irrelevant objects, such as the lesion connecting to (and born through) the border of the image.

### Topological image modification of skin lesion images

#### Smoothing of skin lesion images

**Figure 4:**
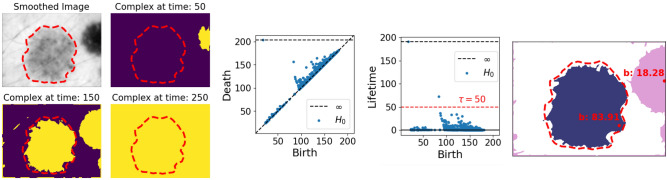
(*Left*) Three complexes $K_t$ at different time steps in the smoothed image ($k=25$). (Middle). The resulting persistence diagram and lifetimes obtained by rotating the diagram. (*Right*) The identified components—those with lifetimes above the thresholds marked by the red striped line—right before their lowest death-time, as well as their birth-pixel and value. Ground truth segmentation borders are marked in red on all images. Lesion image from ISIC 2018^11,12^.

Figure 4 illustrates that image smoothing destructs many irrelevant topological objects, e.g., by blending in outlying clusters of pixels or fine strands of hair with the background. From the persistence diagram of the smoothed image $I'$, we now only deduce one object in the image with a relatively long finite persistence (Fig. 4, Middle). The corresponding component, as well as the component with infinite persistence, are also displayed in Fig. 4 (Right). Note that there is no longer a component corresponding to a cluster of outlying pixels. Furthermore, the component with infinite persistence is now born through a true—although not the relevant—object, instead of the border.

The fact that image smoothing can be regarded as a destructive way of TIM, can also be noted by observing the significant decrease in the number of points in the diagrams in Figs. 3 and 4 after smoothing the image.

#### Border modification of skin lesion images

**Figure 5:**
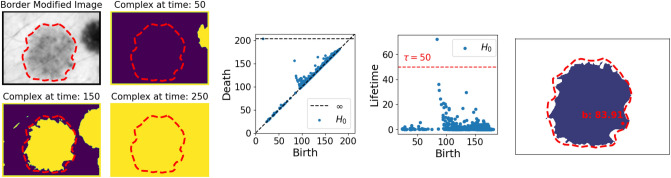
(*Left*) Three complexes $K_t$ at different time steps in the border modified smoothed image ($l=25$). (*Top Right*) The resulting persistence diagram and finite lifetimes obtained by rotating the diagram. (*Bottom Right*) The identified component—the single component with finite lifetimes above the thresholds marked by the red striped line—right before its lowest death-time, as well as its birth-pixel and value. Ground truth segmentation borders are marked in red on all images. Lesion image from ISIC 2018^11,12^.

We showed how image smoothing was able to destruct insignificant and irrelevant topological features in our image. However, depending on the prominence of irrelevant objects, image smoothing is insufficient for this purpose. This is shown in Fig. 4, where the most persisting component actually corresponds to an irrelevant object of the image. Nevertheless, after border modification, we are able to infer the true lesion in the image through the single outlying finite lifetime through Algorithm 1. This is shown in Fig. 5..

We observe that border modification can be regarded both a constructive and destructive way for modifying topological features of an image. On the one hand, we construct a border such that the existence of a corresponding component with infinite persistence is ensured. On the other hand, this process discards all other points in the diagram corresponding to components born through a pixel of this border in the original filtration.

**Figure 6:**
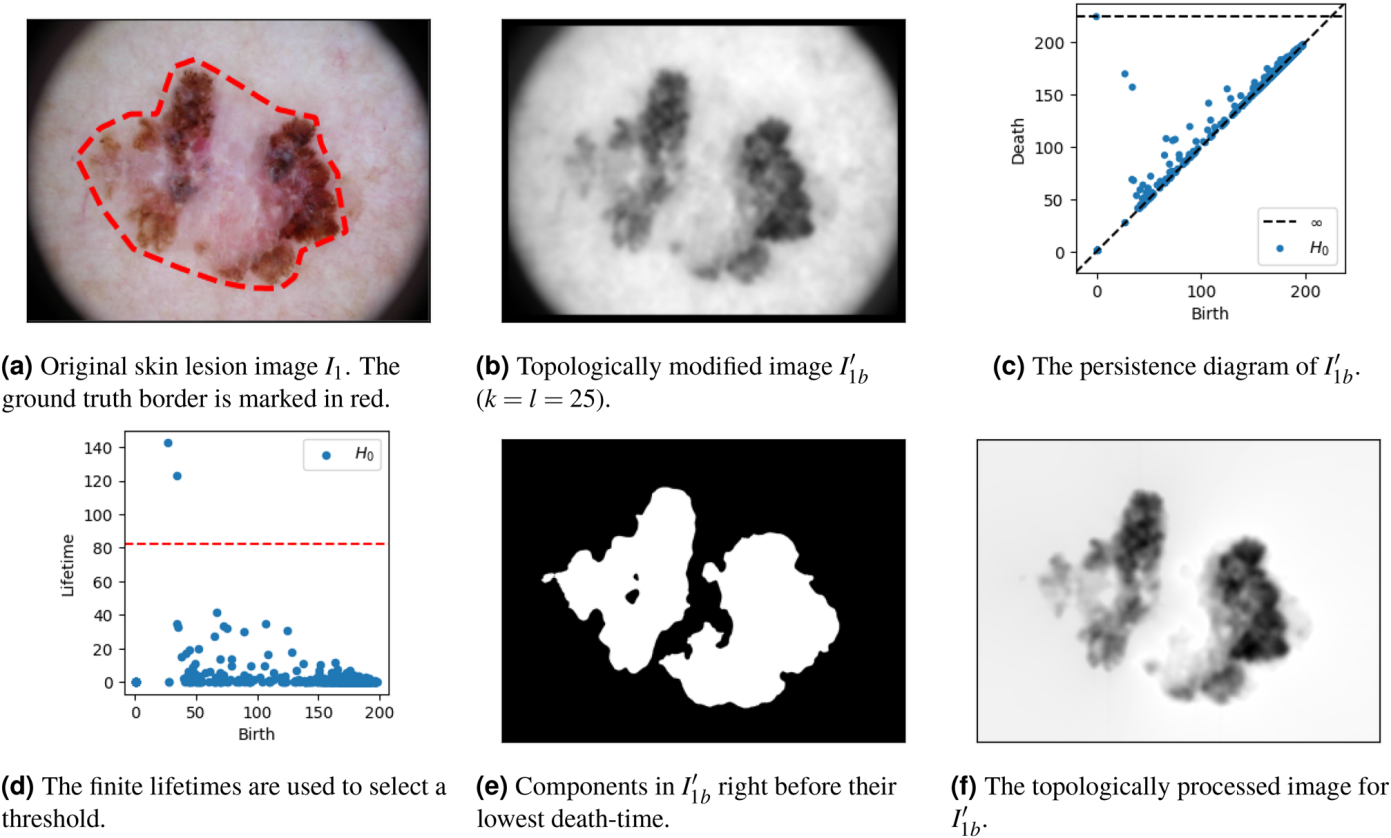
A first example overview of TIP. Lesion image from ISIC 2018^11,12^.

In Summary, the advantages of border modification are the following. First, there is no bias towards the single point with infinite death-time in the persistence diagram (there will always be one for any image). This is especially useful when the birth (pixel) of the corresponding component marks an irrelevant or insignificant object (Fig. 3), or when there are more than one relevant objects identified through topological persistence (Fig. 6). Second, by ensuring relevant objects have finite persistence, we may automatically infer nontrivial thresholds to mark objects in the image through their finite death-times (Algorithm 1). Third, by restricting our analysis to the points of the persistence diagram with finite persistence, any existing outlier detection method can be applied to automatically infer the number of objects displayed by an image based on their persistence.

### Topological image processing of skin lesions

Figure 6 shows the result of topologically processing a skin lesion image displaying a lesion with scarring and regression. This corresponds to the identification of two components through persistent homology (Fig. 6d,e).

**Figure 7:**
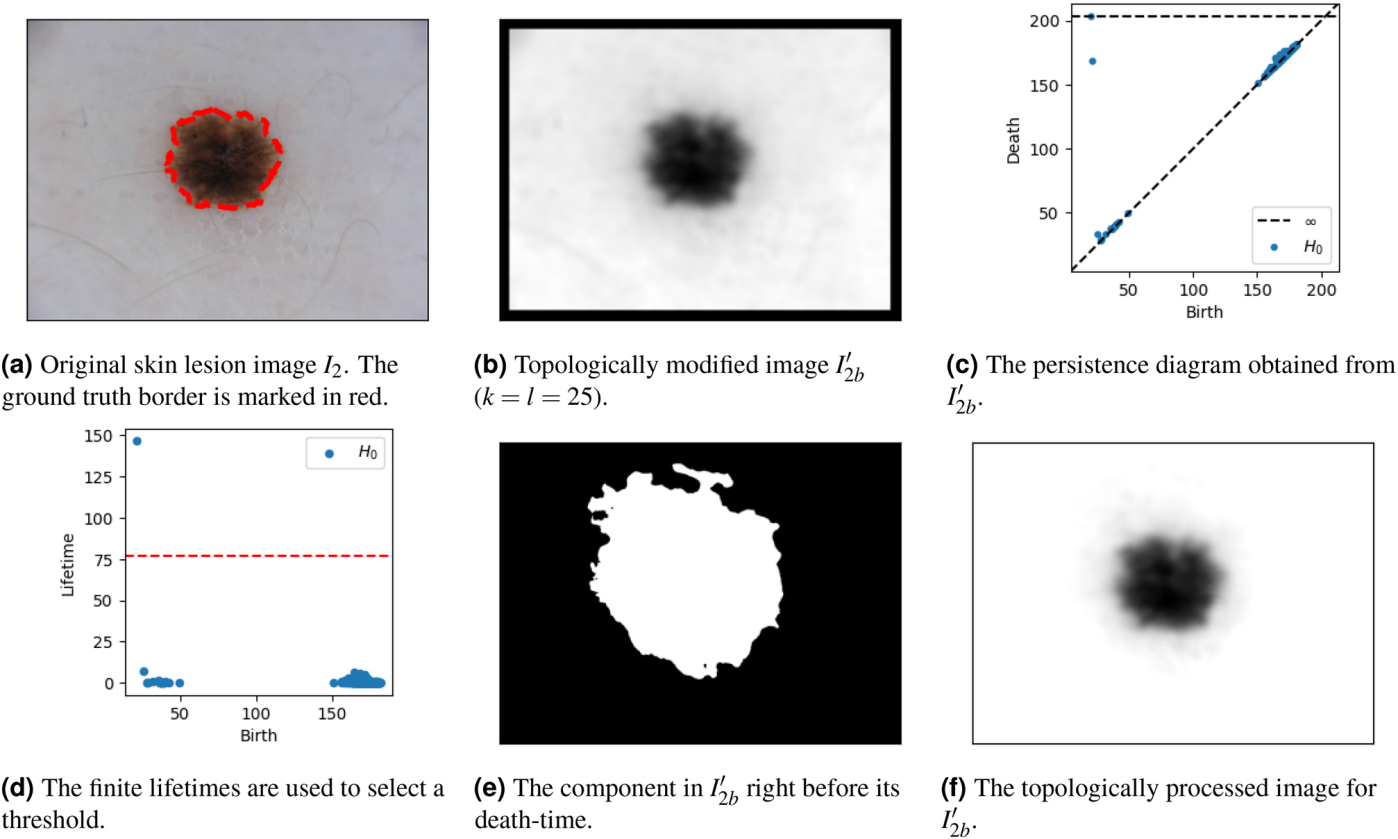
A second example overview of TIP. Lesion image from ISIC 2018^11,12^.

When TIP is used as a first step for segmentation, one may wonder why we conduct our last step. E.g., (the convex hull of) Fig. 6e, which is the output of Algorithm 1, already provides a reasonable segmentation of the lesion. This is because there is no clear gradient between the background and the border of the image. In this case, whenever we start including any background pixel, we rapidly include the majority of background pixels, resulting in the death of the relevant components through the border of the image, guaranteed to be included through TIM. However, when there is a particular gradient in the background that is darker near the object(s) of interest, the identified component(s) will generally include many more pixels than those of the actual object, only marking the area that *includes* the the object(s) of interest. This is illustrated in Fig. 7. Nevertheless, the relevant objects are significantly more highlighted in the topological processed images (Figs. 6f and  7f).

### ISIC 2018 skin lesion segmentation

**Figure 8:**
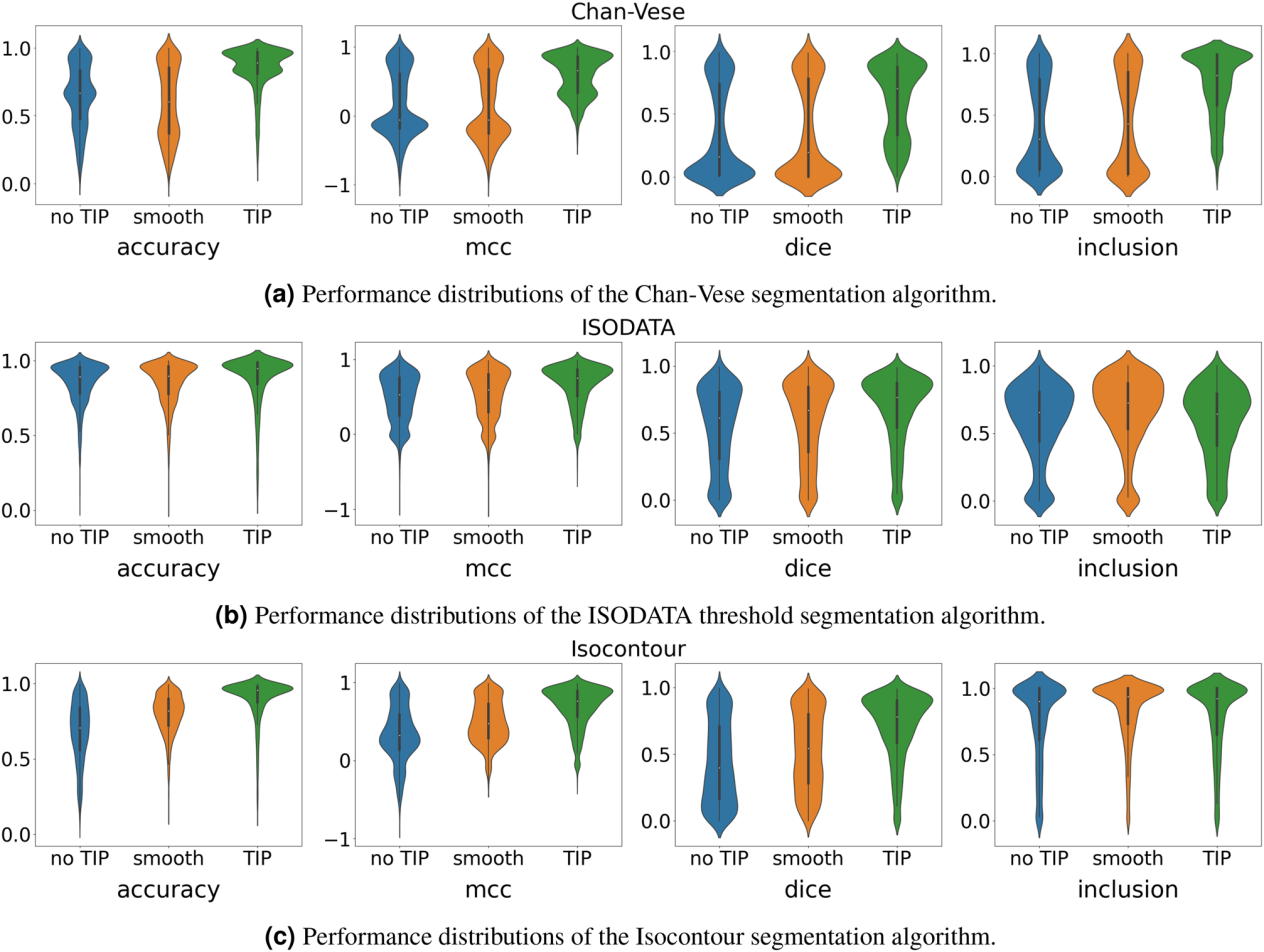
Performance distributions of different segmentations algorithm before and after TIP.

Table 1 shows that the average performance for each of the considered metrics, and each of the considered segmentation methods, before TIP, after smoothing, and after TIP. Figure 8 shows the respective distributions. We consistently observe strong improvements of the segmentations after TIP, with two exception where the inclusion score is better with smoothing only for the ISODATA and Isocontour method. However, as can be deduced from the other metrics, this is accompanied by a large number of false positives. This is exactly as expected from our method, as through TIP, we disregard the irrelevant objects in the image.

**Table 1:** Averaged performances of different segmentation algorithms before TIP, after smoothing, and after TIP.

	Metric	No TIP	Smooth	TIP	Minimal improvement
Chan–Vese	Acc.	0.643	0.599	**0**.**847**	+ 0.204
	Dice	0.346	0.367	**0**.**614**	+ 0.247
	Mcc.	0.152	0.156	**0**.**590**	+ 0.434
	Inc.	0.420	0.454	**0**.**746**	+ 0.292
ISODATA	Acc.	0.850	0.851	**0**.**875**	+ 0.024
	Dice	0.543	0.587	**0**.**672**	+ 0.085
	Mcc.	0.481	0.528	**0**.**657**	+ 0.129
	Inc.	0.587	**0**.**660**	0.584	− 0.076
Isocontour	Acc.	0.680	0.798	**0**.**893**	+ 0.095
	Dice	0.439	0.532	**0**.**704**	+0.172
	Mcc.	0.335	0.492	**0**.**687**	+ 0.195
	Inc.	0.757	**0**.**825**	0.785	− 0.040

The best performance is marked in bold.

**Figure 9:**
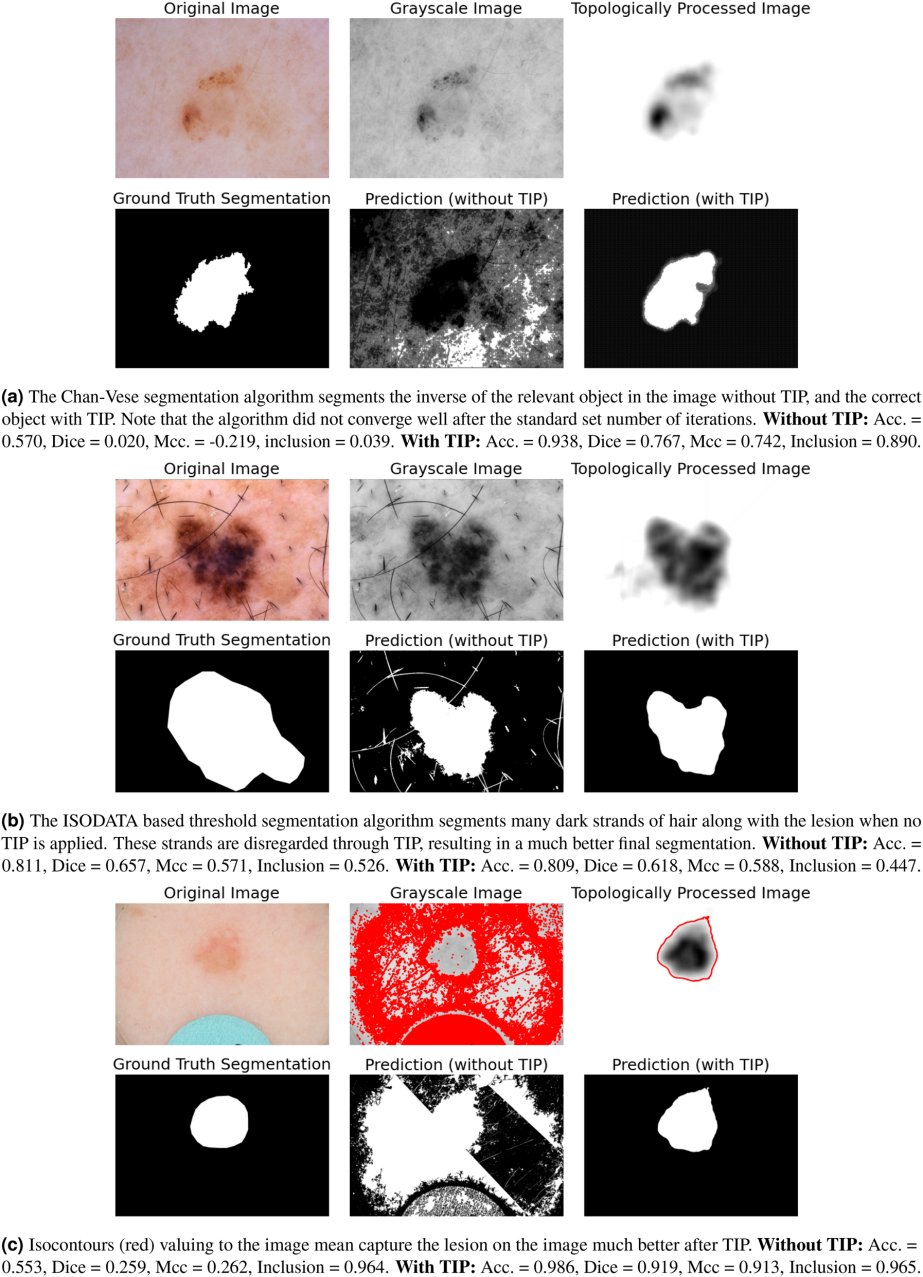
Three examples illustrating how each one of our considered segmentation algorithms benefits from TIP. All lesion images from ISIC 2018^11,12^.

Figure 9a illustrates why the inclusion score most significantly increases for the Chan–Vese segmentation algorithm after TIP. Without TIP, the algorithm appears to often segment the inverse of the actual the lesion. Furthermore, note that in this example, the algorithm did not converge well (after the standard set number of iterations), even when TIP was applied. This results in a checkerboard-like pattern (used to initialize the algorithm) surrounding the actual segmentation, and greatly affects the accuracy, Dice, and Mcc. score. This occurred rather commonly (in approximately 600 topologically processed images), explaining the bimodality of the corresponding distributions in Fig. 8a. Nevertheless, we observe that TIP successfully fulfills its purpose, identifying the lesion in the image and increasing its contrast with the background (Fig. 9a).

Figure 9b illustrates how TIP improves (ISODATA) based threshold segmentation. Without TIP, the darkest parts of the image include the irrelevant strands of hair, and there does not exist any threshold that can be used to include the pixels of the lesion and only those. However, after TIP, the darkest object on the image is the lesion itself, as the strands of hair are disregarded. In this case, (ISODATA) based threshold segmentation does lead to a good result. One may also argue whether the ‘ground truth’ segmentation is actually better than the provided segmentation after TIP in this example. Clearly, TIP significantly improves the segmentation. However, three of the four considered metrics point otherwise (Fig. 9b).

Finally, Fig. 9c illustrates how isocontour based segmentation benefits from TIP. First, after TIP, due to the high contrast between the object and the background, and the homogeneity of the background, it becomes easy to select an appropriate value the isocontours should value to, as the mean of the image values simply suffices. Second, without TIP, there are often many such isocontours, whereas there are commonly only one or a few (correctly) identified contours after TIP (Fig. 9c).

### Other generic unsupervised models

**Figure 10:**
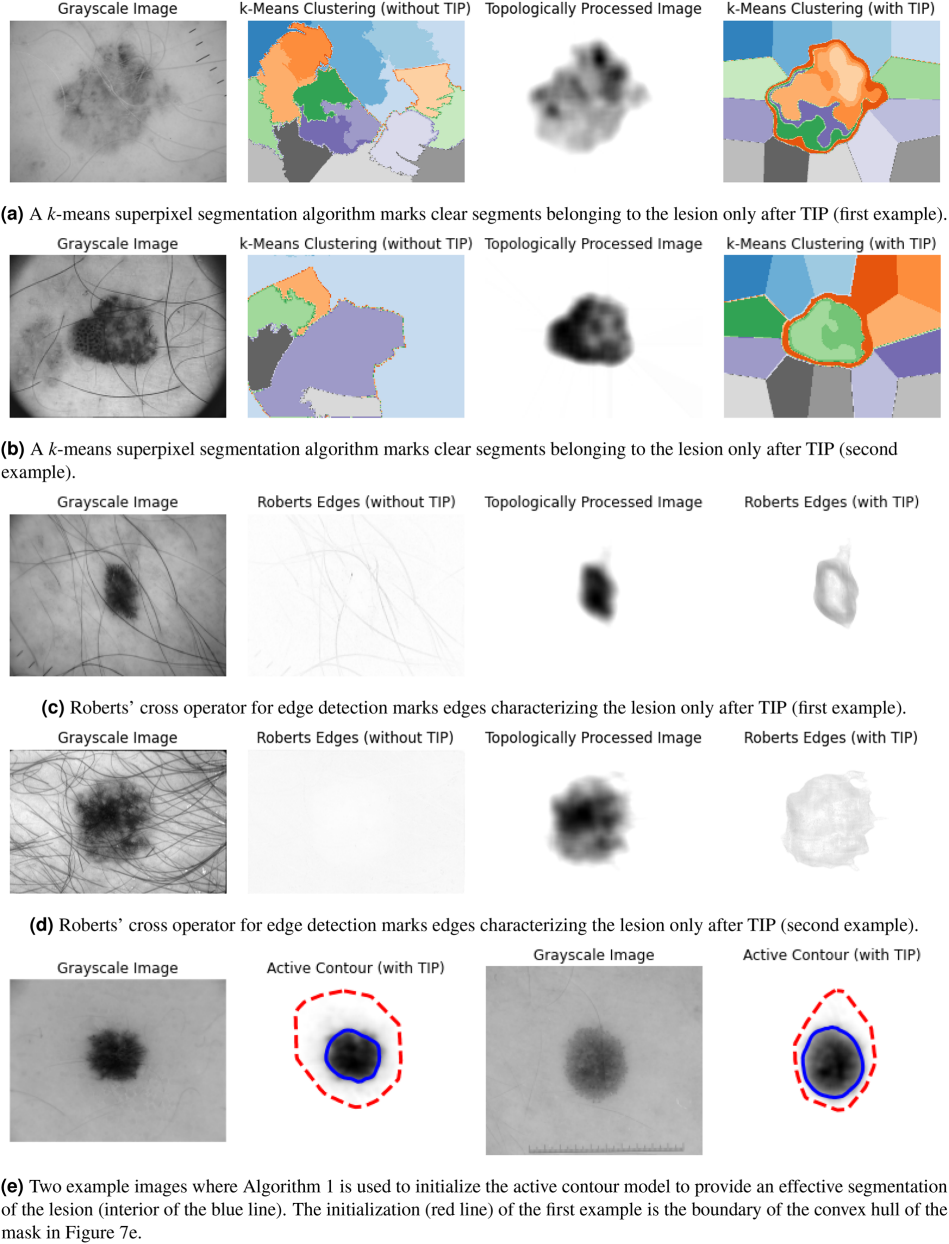
Examples of three other generic and unsupervised models to which TIP provides an effective method to improve their results. All lesion images from ISIC 2018^11,12^.

Figure 10 shows the result of the clustering based segmentation, edge detection, and active contour segmentation algorithm for various skin lesion images. The performance for all images significantly improves after TIP. Note that we did not compare the active contour segmentation before and after TIP, as without TIM, we had no proper way to initialize the contour.

## Discussion

We illustrated how TIP enhances the overall performance of six different and complementary generic models and algorithms to segment skin lesion images in an unsupervised manner. In any of the shown examples, we observe that our method of TIP fulfills its purpose, correctly identifying the relevant objects in the image and increasing their contrast with the background. Interestingly, none of the segmentation methods we considered, showed to be effective for the task of skin lesion segmentation prior to TIP. Furthermore, even in conjunction with TIP, these methods maintained their genericity. Naturally, these methods can be further improved for the particular task of skin lesion segmentation, by trading off their genericity with their performance. E.g., though the ground truth lesions always connected, none of our considered segmentation algorithms necessarily outputs connected segmentations. Their results may be post-processed to accommodate for this restriction.

Since we only considered unsupervised models in our experiments, it may be unrealistic to expect similar performances as the state-of-the-art supervised models, such as convolutional neural networks^16,17^. Rather, we evaluated our work in an unsupervised context to show that TDA using TIM and TIP improves the task of skin lesion segmentation using generic unsupervised segmentation algorithms. It is left to investigate how TIP may enhance the ability to learn in a supervised setting, e.g., as an additional channel to a convolutional neural network based model, or through combined architectures, for either segmentation or classification problems^7^.

As with any method, there are some limitations to our method, both for skin lesion images, as well as for more general applications. We assumed that the relevant objects of our image were darker than the surrounding background, i.e., they have a lower pixel value. In applications where they are actually lighter, one can easily apply our method by constructing the *superlevel filtration* instead of the sublevel filtration, capturing topological information equivalent to the sublevel filtration of the image after negating its pixel values.

**Figure 11:**
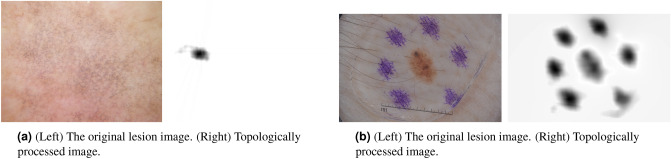
Two example images where our current version of TIM + TIP does not capture the full relevant object and only the relevant object. Both lesion images from ISIC 2018^11,12^.

A more difficult problem is when there is little to no contrast between our object of interest and the background (Fig. 11a). We argue that any learning model, unsupervised or supervised, will find it challenging to correctly identify objects in such images. However, we may automatically recognize these particular types of images based on their persistence diagrams. More specifically, empirical observations show that a stable threshold $\tau$ to identify components may be obtained if the ratio of the width of the largest empty region to the mean width of all empty regions in the persistence diagram is greater than four^28^ . Smaller ratios indicate the absence of contrast between components in images, where it may be difficult to infer the objects through an automatic procedure.

Another difficulty is when more prominent but irrelevant objects are separated from the boundary of the image. e.g, in Fig. 11b, many irrelevant objects, such as the corners of the image, the strands of hair, and the ruler, are destructed in our topologically processed image. However, the surgical marker surrounding the lesion still remain. A different function defining the filtration on the image than the customary grayscale (1), possibly nonlinear in the color channels, that e.g. accounts less or not for the purple colors of the image, may be more appropriate in this case.

The fact that death times commonly occur after the full lesion has been included, prevents us from using persistence diagrams of the topologically modified images for a direct segmentation algorithm. However, in practice, a gradient between the lesion and the background of the image that results in such ‘late’ death-time may also indicate a region of inflammation around the lesion (Fig. 7). Unfortunately, these regions are often disregarded in the ‘ground truth’ (Fig. 7a), and further exploration of this interesting property is required.

Our method works well on images displaying one or few objects of interest on a uniform, noisy, or textured background. This makes skin lesion images an ideal application. For images fully composed of many objects (e.g., a street, cars, houses, trees, ...), other types of models may be more applicable.

## Conclusion

We introduced topological image modification (TIM) as a method for enhancing the ability to extract both relevant and significant topological information from an image. Although image smoothing has been applied in conjunction with persistent homology of images before, its true potential as a destructive topological image modifier has not been studied in detail. Furthermore, we introduced a powerful new method for TIM, i.e., border modification, sensible on three different levels. First, we discard all bias towards the single component with infinite persistence. Second, we may automatically and consistently separate objects from the background through their finite death-times (Algorithm 1). Third, any outlier detection method to automatically infer the number of components through the persistence diagram of the image becomes well-defined.

We introduced the concept of, as well as a new method for topological image processing (TIP). We showed how this method significantly increased the performance of six different generic and unsupervised models and algorithms through a wide variety of of skin lesion images from ISIC 2018. Furthermore, this increase in performance was extensively quantified on all 2594 skin lesion images, for the three algorithms that led to a straightforward binary segmentation method before and after TIP. Though this is a very domain-specific application, our method for TIP is very generic, resting on the assumptions that outliers can be destructed through smoothing the image, and that the relevant object(s) are away from the border of the image.

Finally, the idea behind TIM—altering the topological properties of an image to prevent the detection of irrelevant or insignificant objects—is very generic as well. Hence, a wide variety of topological modifiers, as well as new applications of TIP to supervised learning and domains other than (segmenting) skin lesions are yet to be discovered.
